# Decreased Pre-existing Ad5 Capsid and Ad35 Neutralizing Antibodies Increase HIV-1 Infection Risk in the Step Trial Independent of Vaccination

**DOI:** 10.1371/journal.pone.0033969

**Published:** 2012-04-04

**Authors:** Cheng Cheng, LingShu Wang, Jason G. D. Gall, Martha Nason, Richard M. Schwartz, M. Juliana McElrath, Steven C. DeRosa, John Hural, Lawrence Corey, Susan P. Buchbinder, Gary J. Nabel

**Affiliations:** 1 Vaccine Research Center, National Institute of Allergy and Infectious Diseases, National Insititutes of Health, Bethesda, Maryland, United States of America; 2 Biostatistics Research Branch, National Institute of Allergy and Infectious Disease, National Institutes of Health, Bethesda, Maryland, United States of America; 3 GenVec, Inc., Gaithersburg, Maryland, United States of America; 4 Vaccine and Infectious Disease Division, Fred Hutchinson Cancer Research Center, Seattle, Washington, United States of America; 5 HIV Vaccine Trials Network, Seattle, Washington, United States of America; 6 HIV Research Section, San Francisco Department of Public Health, San Francisco, California, United States of America; French National Centre for Scientific Research, France

## Abstract

**Background:**

The Step trial raised the possibility that uncircumcised men with pre-existing Ad5 neutralizing antibodies carried an increased risk of HIV infection after vaccination. Thus, understanding Ad seropositivity in humans is important to the development of an AIDS vaccine. Here, we analyze the impact of different Ad5-specific neutralizing antibodies on immune function and clinical outcome.

**Methods and Findings:**

Ad seropositivity in the Step trial volunteers was analyzed using chimeric rAd5/35 vectors to characterize their specificity for Ad5 fiber and non-fiber external (capsid) proteins. Immune responses and HIV seropositivity were correlated with the specificity of Ad5-neutralizing antibodies. Neutralizing antibodies induced by the vaccine in Ad5 seronegative subjects were directed preferentially to Ad5 capsid proteins, although some fiber-neutralizing antibodies could be detected. Pre-vaccination Ad5 serostatus did not affect the capsid-directed response after three vaccinations. In contrast, anti-fiber antibody titers were significantly higher in volunteers who were Ad5 seropositive prior to vaccination. Those Ad5 seropositive subjects who generated anti-capsid responses showed a marked reduction in vaccine-induced CD8 responses. Unexpectedly, anti-vector immunity differed qualitatively in Ad5 seropositive participants who became HIV-1 infected compared to uninfected case controls; Ad5 seropositive participants who later acquired HIV had lower neutralizing antibodies to capsid. Moreover, Ad35 seropositivity was decreased in HIV-infected subjects compared with uninfected case controls, while seroprevalence for other serotypes including Ad14, Ad28 and Ad41 was similar in both groups.

**Conclusions:**

Together, these findings suggest that the case subjects were less immunologically responsive prior to infection. Subjects infected during the Step trial had qualitative differences in immunity that increased their risk of HIV-1 infection independent of vaccination.

## Introduction

The Step study was a phase IIB clinical trial designed to test a recombinant adenovirus 5 (rAd5) based HIV vaccine in 3000 participants who were seronegative or seropositive for pre-existing Ad5 neutralizing antibodies. An interim analysis of Step study participants revealed no beneficial effect of vaccination on HIV viral load or acquisition of infection in vaccinated individuals vs. placebo controls and also showed a trend towards increased HIV infection after vaccination in uncircumcised men with pre-existing Ad5 neutralizing antibodies [1], [2]. Thus, understanding the nature and immune effects of Ad5 seropositivity in humans is important for the development of rAd-based vaccines against AIDS.

Ad5 viruses are non-enveloped virions composed of fiber and two major capsid proteins, hexon and penton base (penton), all of which are exposed on the virion surface. Neutralizing antibodies to these proteins mediate viral inactivation. Specifically, anti-fiber antibodies can prevent viral entry, and antibodies to other capsid proteins can also interfere with viral uptake and viral endosomal escape during cell infection [3]. These antibodies also synergize with each other to achieve maximum viral neutralization [3], [4]. Prior research on pre-existing neutralizing antibodies to Ad5 in participants of this trial established the titer of neutralizing antibodies to the whole Ad5 virion [1], [2]. We have previously developed chimeric rAd vectors that can be used to analyze the role of specific neutralizing antibodies against individual viral proteins [5]. The panel of rAd reporters included two chimeric vectors and the parental, unmodified rAd5 and rAd35 vectors. The rAd35 vector was used as the control backbone because human sera rarely neutralize this serotype at high titers and participants seropositive for this control backbone confound the analysis and must be excluded from the subsequent immune analysis. The two chimeric vectors are rAd5 Fiber (F)35 and rAd35 F5 vectors, with the fiber of Ad35 grafted onto rAd5 or with the fiber of Ad5 grafted onto rAd35, respectively. rAd5 F35 vectors were used to detect neutralizing antibodies to Ad5 capsid and rAd35 F5 vectors were used to detect anti-Ad5 fiber neutralizing antibodies. The properties of these reporter vectors reflect those found on the native Ad serotypes and therefore can be used to detect the specificity and function of the respective viral components and analyze anti-Ad immunity in humans [5]. Analysis of sera from participants in the HVTN 204 trial has shown that the distribution of individual capsid-specific neutralizing antibodies varies among these sera, and the pre-existing fiber-specific neutralizing antibodies correlated with both a reduced response rate and the magnitude of anti-Gag T-cell responses induced by DNA prime and rAd5 boost HIV vaccines [5].

The major objectives of this study were to determine the specificity of neutralizing antibodies (Nab) to Ad5 fiber and major capsid proteins (hexon and penton base) and the potential association with immunogenicity and risk of infection in participants of the Step trial. This analysis provided an opportunity to determine whether natural infection by adenovirus stimulated neutralizing antibodies to different Ad5 viral proteins than rAd5 vector vaccination alone and whether they might exert differential effects on vaccine immunogenicity that could affect susceptibility to HIV infection. Finally, we sought to determine whether participants who became infected despite HIV vaccination developed anti-Ad5neutralizing antibodies with distinct specificities. An understanding of the nature of anti-Ad5 immunity and its effect on the Step trial has implications for the design of next generation vectors for AIDS vaccines.

## Results

### MRK Ad5 vaccination induced neutralizing antibodies to the Ad5 fiber protein and capsid proteins in Ad5 seronegative subjects

**Figure 1:**
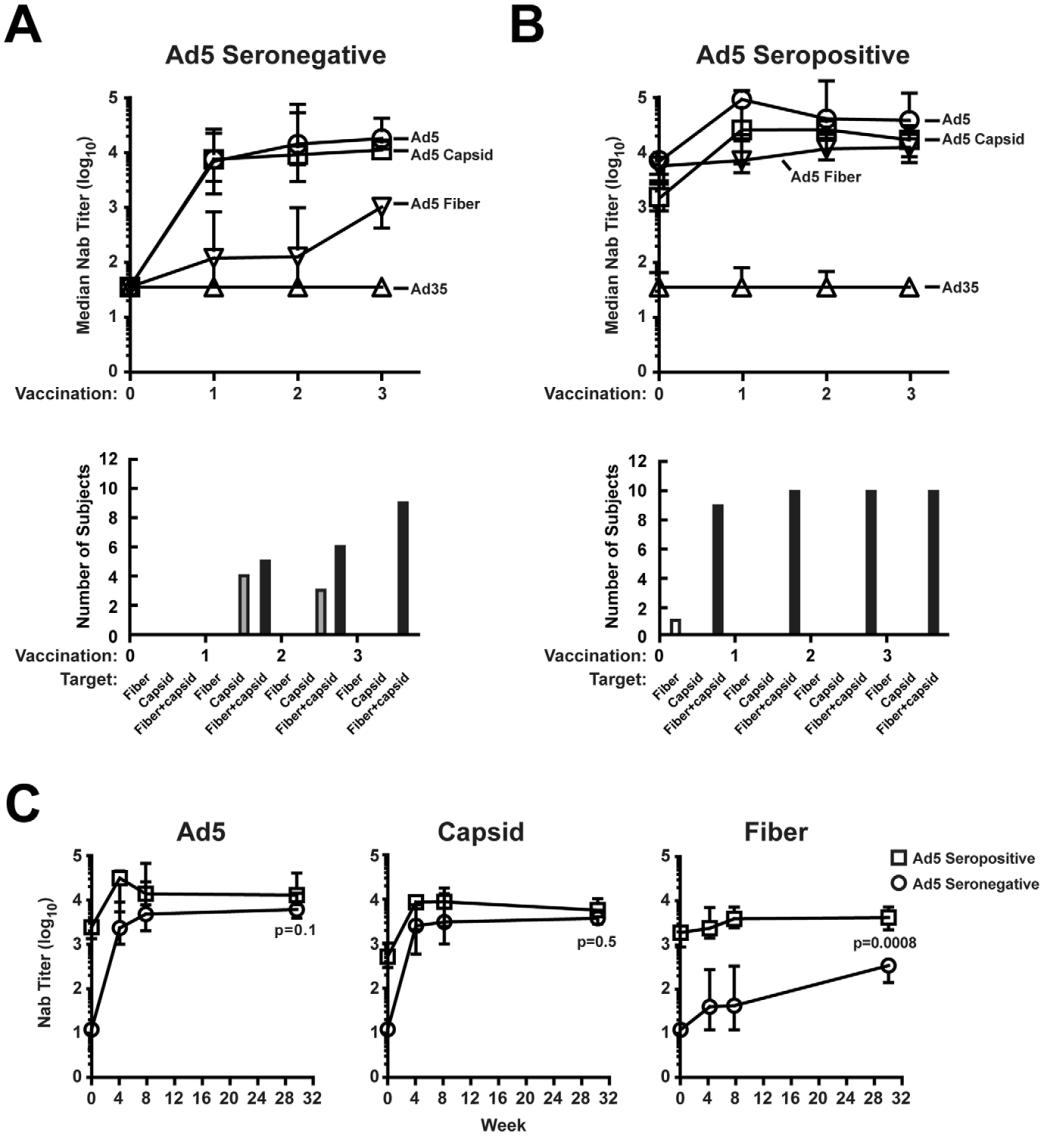
MRKAd5 HIV vaccine generated neutralizing antibodies to Ad5 capsid proteins more efficiently than to Ad5 fiber. The titers of Nab to Ad5, Ad5 F35, Ad35 and Ad35 F5 were determined in Ad5 seronegatives and Ad5 seropositives following vaccinations. The minimum detectable titer is at 1∶12 serum dilution and the maximum titer is at the end point dilution to achieve 90% neutralization of the vector. The development of Nab in baseline seronegatives (**A**) or seropositives (**B**) at 4 weeks after each vaccination was also shown. **A** (lower panel) and **B** (lower panel) show the Nab targets in these sera, excluding sera samples with high anti-Ad35 Nab titers which confound the categorization of the Nab target. (**C**) Direct comparison of the titer of Nabs to Ad5, or Ad5 capsid, or Ad5 fiber between baseline seronegatives and seropositives. Day 0: pre-vaccination; Week 4: 4 weeks after the first vaccination; Week 8: 4 weeks after the second vaccination; Week 30: 4 weeks after the third vaccination.

To determine whether vaccine recipients developed antibodies to Ad5 after vaccination, we analyzed sera from 22 participants prior to vaccination and at week 4, 8, and 30. At day 0, pre-existing Nabs to Ad5 were not detected in 11 Ad5 seronegative participants (Figure 1A, upper panel) and the sera from two participants were excluded from subsequent analysis because anti-Ad35 Nab were detected, which prevented categorization of the anti-Ad5 Nab activities based on the two chimeric reporter vectors that utilized Ad35 components. At week 4 (after the first vaccination), all of the remaining 9 participants became Ad5 seropositive, and their Nabs were directed to capsid proteins other than fiber in 4 participants or to both fiber and capsid in the other 5 participants (Figure 1A; lower panel, capsid, fiber + capsid). The specificity of this Nab response changed over the course of vaccination. By the third vaccination at week 30, all 9 participants developed Nabs to both fiber and capsid proteins (Figure 1A, lower panel, 9 participants with undetectable anti-Ad35 neutralizing antibodies were analyzed). These results indicate that vaccination readily generated high titers of Nab to the capsid and weakly induced neutralizing humoral immunity solely to the fiber. This trend was also confirmed in the median titer generated by vaccination (Figure 1A, upper panel). While a high titer of Nab to the capsid was generated after a single vaccination, there was a low titer of anti-fiber Nab present in these participants, and it remained at least 10-fold lower than anti-capsid antibodies despite a gradual increase of Nab to the fiber after vaccination.

### Characterization of neutralizing antibodies to Ad5 fiber and capsid proteins in the presence of pre-existing Ad5 immunity

Pre-existing immunity to Ad5 was documented in 11 Ad5 seropositive participants whose sera contained Nabs to Ad5 prior to the first immunization. In contrast to the vaccine-induced Nabs to Ad5 elicited by vaccination in the seronegative group, neutralizing activity directed to the fiber was detected in all 11 pre-immune vaccine sera (Figure 1B, upper panel). One participant was excluded from further analysis due to high titers of anti-Ad35 Nabs detected at week 4. Of the remaining 10 participants, one subject's Nabs were directed exclusively to the fiber, while the other 9 participants had Nabs to both fiber and capsid prior to vaccination. After the first immunization, all sera showed an increase in the neutralization titer directed to both fiber and capsid (Figure 1B, lower panel, 10 participants with undetectable anti-Ad35 neutralizing antibodies were analyzed). Comparing the Nab titers of seropositives with seronegatives, we found, as expected, that the titers of anti-Ad5, anti-Ad5 capsid, and anti-Ad5 fiber Nab were all higher in seropositives compared to the seronegatives (Figure 1C, day 0). After the first vaccination, the anti-capsid titers increased more than the anti-fiber response (Figure 1C, week 8). After a second vaccination, the magnitude of this difference was reduced; by week 30, there was no significant difference in terms of Nab titers to Ad5 or to Ad5 capsid. On the other hand, the titer of Nab to Ad5 fiber in the seronegative group was significantly lower than in the seropositive groups at all time points (Figure 1C; Fiber, p<0.05).

### Characterization of pre-existing neutralizing antibody to fiber and capsid proteins in HIV-uninfected participants

**Figure 2:**
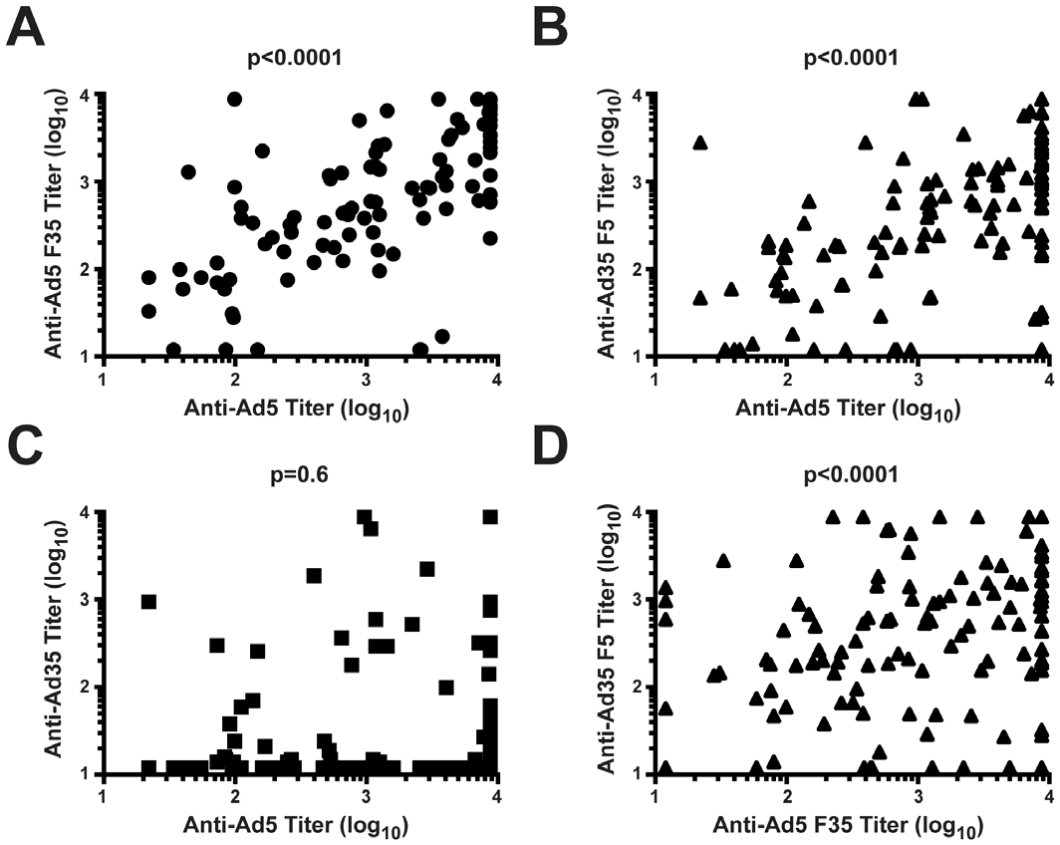
The titers of Nab to Ad5, Ad5 F35, Ad35, and Ad35 F5 were determined in pre-vaccination sera from 128 HIV-uninfected participants, and the correlation between the titers of Nab to each vector were analyzed with p<0.05 suggesting a significant correlation (A, B, C, D).

**Figure 3:**
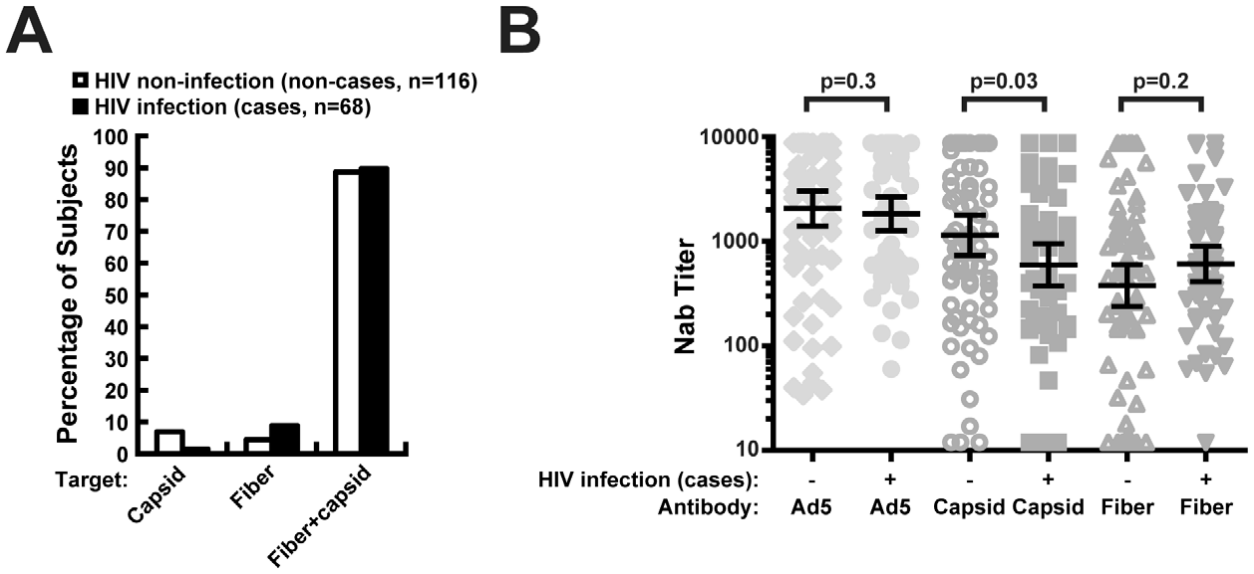
The neutralizing targets were determined for 116 out of the 128 HIV-uninfected participants and 68 out of the 70 HIV-infected participants after excluding participants with high titers of anti-Ad35 Nab which confound categorization. (**A**). Pre-existing Nab titers in HIV-uninfected (n = 75) and HIV-infected (n = 57) participants were compared among participants with low anti-Ad35 titers (titer<12) (**B**). Geometric mean and 95% CI was shown in the background of Nab titers in each individual.

We examined 179 sera at day 0 from HIV-uninfected subjects. Of these, 51 serum samples were classified as Ad5 seronegative (titers <18). The remaining 128 samples were Ad seropositive (titers ≥18). These results were largely consistent with testing by the HVTN laboratory, although 9 seronegatives in the HVTN assay were determined to be seropositive with low titers due to the sensitivity of our assay to detect low titer samples. When the Ad5 seropositive group was further analyzed to determine the specificity of their responses, we found a correlation between anti-Ad5 titers with either the anti-capsid (Figure 2A; Ad5 F35 Spearman correlation r = 0.79) or the anti-fiber (Figure 2B; Ad35 F5 Nab titer, Spearman correlation r = 0.63), and a correlation between anti-Ad5 capsid (Ad5 F35) and anti-Ad5 fiber (Ad35 F5, Figure 2D Spearman correlation r = 0.43) based on a nonparametric correlation (Spearman) analysis (Figure 2, A, B, and D, p<0.0001),while there was no correlation between anti-Ad5 and anti-Ad35 Nab titers (Spearman correlation r = −0.04, p>0.05, Figure 2C), documenting the serotype specificity of the Ad5 seropositive group. After excluding 12 samples due to anti-Ad35 titers, we were able to characterize the neutralization targets in 116 samples. Nab to both Ad5 fiber and Ad5 capsid were detected in a majority of the samples (89%), while Nab to capsid only or fiber only was detected exclusively in a small portion of the samples (Figure 3A, 7% and 4%, respectively).

### Effect of pre-existing Ad5 immunity on vaccine-induced immune responses to HIV antigens in the HIV-uninfected subjects

We next compared the magnitude of IFN-γ and/or IL-2 secreting CD4^+^ or CD8^+^ T cells specific to HIV antigens between the seropositive and seronegative groups with data from the HVTN immunology laboratory from the 179 participants who were all in the vaccine group at week 30. The presence of pre-existing Nab to either Ad5 (Table 1), the capsid (Table 2), or the fiber (Table 3) all correlated with reduced vaccine-induced immunogenicity, especially evident in the lower CD8 T-cell frequencies recognizing Gag, Pol, and Nef. As shown in Table 1, for example, in the Ad5 seronegative group, the median HIV Gag-specific CD8 frequency was 0.088%. In contrast, in the Ad5 seropositive group, the response was significantly reduced to 0.017% (p<0.0001). This reduction in the immune response correlated more closely with the Nab directed to Ad5 fiber (Table 3) than capsid (Table 2) based on the p-value when CD8 immune responses in seronegatives and seropositives were compared. In contrast, CD4 responses were less affected by Ad5 seropositivity, especially those directed to Gag and Pol. The anti-Gag and anti-Pol-specific CD4 responses were similar in both Ad5 seropositive and seronegative groups (p>0.05, Table 1). CD4 responses directed to Nef were somewhat reduced in the Ad5 seropositive group (p<0.05, Table 1), suggesting that immune effects were dependent to some extent on the antigen.

**Table 1:** Pre-existing anti-Ad5 neutralizing antibodies reduced immune responses generated by MRKAd5 HIV-1 gag/pol/nef vaccine, especially HIV-specific CD8+ immune responses.

		IFN-γ^+^ and/or IL-2^+^ Median Frequency (%)		
Specific T cells		Ad5 seronegative (n = 36–39)	Ad5 seropositive (n = 66–75)	p value
Gag	CD4	0.064 (0.041–0.083)	0.060 (0.032–0.099)	0.4
	CD8	0.088 (0.051–0.236)	0.017 (0.000–0.090)	<0.0001***
Nef	CD4	0.040 (0.015–0.071)	0.018 (0.003–0.036)	0.007***
	CD8	0.271 (0.121–0.819)	0.080 (0.009–0.307)	0.0005***
Pol	CD4	0.017 (0.003–0.041)	0.012 (0.000–0.030)	0.3
	CD8	0.112 (0.044–0.316)	0.042 (0.009–0.106)	0.001**

Median (25%–75% percentile),

* : p<0.05;

** : p<0.01,

*** : p<0.001.

**Table 2:** Pre-existing anti-Ad5 capsid neutralizing antibodies reduced immune responses generated by MRKAd5 HIV-1 gag/pol/nef vaccine, especially HIV-specific CD8+ immune responses.

		IFN-γ^+^ and/or IL-2^+^ Median Frequency (%)		
Specific T cells		Ad 5 capsid seronegative (n = 41–44)	Ad5 capsid seropositive (n = 66–74)	p value
Gag	CD4	0.064 (0.037–0.078)	0.063 (0.033–0.106)	0.9
	CD8	0.067 (0.027–0.209)	0.016 (0.000–0.103)	0.001**
Nef	CD4	0.037 (0.011–0.071)	0.023 (0.004–0.038)	0.03*
	CD8	0.215 (0.087–0.717)	0.088 (0.008–0.430)	0.01*
Pol	CD4	0.014 (0.000–0.040)	0.014 (0.002–0.036)	0.9
	CD8	0.101 (0.032–0.228)	0.046 (0.011–0.121)	0.03*

Median (25%–75% percentile),

* : p<0.05;

** : p<0.01,

*** : p<0.001.

**Table 3:** Pre-existing anti-Ad5 fiber neutralizing antibodies reduced immune responses generated by MRKAd5 HIV-1 gag/pol/nef vaccine, especially HIV specific CD8+ immune responses.

		IFN-γ^+^ or IL-2^+^ Median Frequency (%)		
Specific T cells		Ad 5 fiber seronegative (n = 43–47)	Ad5 fiber seropositive (n = 59–69)	p value
Gag	CD4	0.064 (0.044–0.081)	0.064 (0.031–0.108)	0.8
	CD8	0.088 (0.027–0.237)	0.017 (0.000–0.075)	0.0002***
Nef	CD4	0.034 (0.011–0.069)	0.024 (0.003–0.041)	0.09
	CD8	0.271 (0.092–0.816)	0.081 (0.010–0.297)	0.002**
Pol	CD4	0.014 (0.000–0.040)	0.014 (0.002–0.036)	0.8
	CD8	0.103 (0.044–0.300)	0.034 (0.009–0.106)	0.0009***

Median (25%–75% percentile),

* : p<0.05;

** : p<0.01,

*** : p<0.001.

### Characterization of Ad neutralizing antibodies in HIV-infected subjects

In the trial, at the time of this analysis, a total of 71 participants acquired HIV infection after vaccination in the placebo and vaccine groups. To determine whether susceptibility to HIV infection was related to the specificity of pre-existing anti-Ad5 Nabs, we evaluated the specificity of the response and their effects on vaccine immunogenicity. Sera from 70 of these individuals were pre-existing seropositive for Ad5, of which 26 sera were derived from the placebo group and 44 from vaccinees. There was no significant difference between the placebo and vaccine groups in the titer of Nab to Ad5, Ad5 F35 (capsid), Ad35, or Ad35 F5 (fiber) (Figure S1, p>0.05). The majority of the sera contained both anti-capsid and anti-fiber Nab (Figure 3A). These included 61 out of 68 subjects (Figure 3A, 90%), with two sera excluded due to high anti-Ad35 Nab titer. These findings were not unexpected because of the high rates of pre-existing Ad5 immunity within this subgroup. Most of the remaining sera contained anti-fiber Nab exclusively (9%). When the titer of anti-Ad5 (p = 0.8), capsid (p = 0.5), or fiber (p = 0.4) responses were compared between circumcised (n = 30) and non-circumcised (n = 31) males, no significant difference between these two groups was evident.

To reveal any unique features of anti-Ad5 Nab in HIV-infected subjects, we compared the titer of pre-existing Ad5 Nab in uninfected and infected Ad5 seropositive groups, including placebo and vaccine groups, as well as Ad35 seronegative participants. This comparison revealed no significant difference in the titer of anti-Ad5 in these two groups (Figure 3B). At the same time, a significantly lower anti-Ad5 capsid Nab titer (Figure 3B, geometric mean 597 vs. 1149, p = 0.03) was observed in HIV-infected individuals. This trend was also reflected in the distribution of Ad5 Nab neutralizing targets as shown in Figure 3A. For example, while Nab to capsid only was detected in a small portion of HIV-uninfected subjects (7%), it was seen even more rarely in HIV-infected participants (Figure 3A, 1%). We also analyzed the HIV uninfected and infected Ad5 seropositive groups based on Ad35 seropositivity. In the HIV-infected group, 13 participants had pre-existing Nab to anti-Ad35 (Table 4, 19% of the total). In contrast, 53 (41%) of the 128 HIV-uninfected participants were Ad35 seropositive. The Ad35 seroprevalence was therefore significantly lower in the HIV-infected group compared to the HIV-uninfected group (p = 0.002). On the other hand, the seroprevalence for Ad14 (serotype B), Ad28 (serotype D) and Ad41 (serotype F) in these two groups were not significantly different (Table 5). These findings suggested that these HIV-susceptible individuals had a unique immune profile to Ad5 capsid and Ad35, both prior to vaccination and afterwards, that may have predisposed them to HIV-1 infection.

**Table 4:** Seroprevalence of Ad35 in HIV-infected, Ad5 seropositive participants is lower compared to that in HIV-uninfected, Ad5 seropositive participants.

	HIV-infected	HIV-uninfected
**Ad35 seropositive**	13*	53*
**Ad35 seronegative**	57*	75*
**Total (% Ad35 seropositive)**	70 (19%)	128 (41%)

* values used for Fisher's exact test, p = 0.002.

**Table 5:** Seroprevalence of Ad14, Ad28 and Ad41 in HIV-infected, Ad5 seropositive and HIV-uninfected, Ad5 seropositive participants is similar.

	HIV-infected	HIV-uninfected	P*
**Ad14 seroprevalence**	51%	56%	0.5
**Ad28 seroprevalence**	58%	69%	0.2
**Ad41 seroprevalence**	94%	99%	0.1

* Fisher's exact test.

## Discussion

rAd vectors are widely used in the field of gene therapy and vaccination due to their efficiency for gene delivery and ability to stimulate cellular and humoral immune responses. However, the potential effects of pre-existing immunity to Ad on vector delivery and vaccine efficacy raise complications for clinical implementation. In the Phase IIB Step trial, the high rates of infection in the vaccine group, along with the lack of protective efficacy, led to termination of the study. These concerns highlight the need to understand the role of anti-vector immunity and its impact on efficacy. Such pre-existing immunity against the vector can blunt the efficiency of gene delivery and may cause side effects on disease outcomes. Despite substantial effort, no causal link has been established between Ad5 seropositivity, the MRKAd5 vaccination, and the risk of HIV acquisition, although retrospective analyses suggest a higher risk of infection after vaccination in Ad5 seropositive, uncircumcised participants [1].

Several hypotheses have been proposed to explain this trend. One is that the rAd5 vector may activate pre-existing Ad5-specific CD4 T cells to express HIV co-receptors in Ad5 seropositives [6]. However, it was shown that there is no correlation between Ad5 serostatus and the presence of anti-Ad5 T-cell responses in humans [7], [8]. Furthermore, there was no elevated, long lasting reactivation of Ad5-specific CD4+ T-cell responses after rAd5 vaccination in seropositive populations [7], [8]. Therefore, it is unlikely that Ad5-specific CD4+ T cells reactivated by vaccination resulted in increased HIV targeting cells in the systemic compartment. Recent studies in monkeys and in Step participants have also suggested that there is no greater reactivation or trafficking of CD4+ T cells to mucosal compartments in Ad5 seropositives compared to Ad5 seronegatives post-vaccination [9]. Another hypothesis is that anti-Ad5 neutralizing antibodies can form immune complexes with rAd5 vectors and dendritic cells which can enhance HIV infection in T cells. Although this possibility was demonstrated *in vitro* [10], it is unknown how it correlates to the responses found in the high risk group of the Step trial.

In this study, we analyzed the specificity of anti-Ad5 Nab in Step trial participants and determined whether differences in specificity correlated with Ad5 seropositivity and risk for HIV infection. The seroprevalence reported here for Ad5 is higher than assayed by HVTN as 9 out of the 60 seronegatives were seropositive by our assay. This is likely due to the use of EDTA for harvesting cells rather than trypsin, which can cleave CAR and compromise physiological functions that require intact CAR protein [11]. Trypsin may also cleave other cellular receptors, including integrins that affect virus uptake [12]. Although the proteolysis of CAR by trypsin is limited to one cleavage site, trypsin treatment may affect the sensitivity of the assay to detect neutralizing antibodies to other rAd that use trypsin-sensitive cellular receptors [13]. The results show that the MRKAd5 HIV vaccine efficiently converted all the Ad5 seronegative participants to seropositivity after a single vaccination. Thus, pre-vaccine serostatus was not maintained, even at four weeks after the first vaccination. Our data also suggest that vaccination induced Nab to the capsid of Ad5 more efficiently than to the fiber. This result is consistent with a previous study that showed differences in the specificity of Ad5 Nab from natural infection and vaccination with rAd5 vectors [5]. Vaccination generated Nab to Ad5 capsid more efficiently than to fiber, while natural infection generated Nab to both Ad5 fiber and capsid. This may be due to the fact that the rAd vaccines are non-replicating and contain more capsid proteins than fiber proteins. In the Step trial, we show here that one vaccination was sufficient to convert all vaccinees to Ad5 capsid seropositivity, while three vaccinations converted them all to Ad5 fiber seropositivity. Therefore, pre-vaccination serostatus affected post-vaccination neutralizing antibody profiles and the quantity of anti-Ad5 fiber Nabs. There is no evidence to suggest that such quantitative differences in the titers of anti-Ad5 fiber Nab between these two groups correlated with HIV infection risk in the participants.

There were no significant differences in the titer of Ad5 neutralizing antibodies between HIV-infected and uninfected participants, and no significant differences among HIV-infected participants in the vaccine and placebo groups (Figure S1). These data are consistent with a recent report showing that anti-Ad5 neutralizing antibody is not associated with risk of HIV infection in the absence of vaccination [14]. On the other hand, further analysis of HIV-infected participants showed that their sera tend to contain lower anti-Ad5 capsid Nabs compared to that detected in uninfected participants. It seems that anti-vector immunity differed qualitatively in participants who acquired HIV infection compared to those who did not.

This finding highlights the fundamental differences in these participants in terms of how their immune systems recognize multiple components of viral antigens. Although our assay did not distinguish anti-hexon and anti-penton antibodies targeting the viral capsid, previous studies have demonstrated that anti-hexon and anti-fiber antibodies play a major role in mediating neutralization in Ad5 seropositive people and subjects vaccinated with rAd5 vectors [5], [15]–[17]. Anti-penton antibodies have also been detected in cancer patients receiving rAd5 therapies [18]. Therefore, the anti-capsid antibodies documented here are likely composed of both anti-hexon and anti-penton antibodies, with anti-hexon antibodies as the major contributor to neutralization. As shown here, multiple neutralizing epitopes targeted by the immune system exist for adenoviruses. These epitopes can be serotype-specific or located in conserved regions among adenoviridae. It has been documented that Ad fiber-specific neutralizing antibodies are more serotype-specific and Ad capsid-targeted antibodies are group-specific with a broader spectrum [19], [20]. It seems that HIV-infected participants had lower baseline Ad5 capsid neutralizing antibodies compared to HIV-uninfected participants. It is interesting to note that 41% were Ad35 seropositive among the Ad5 seropositive HIV-uninfected participants compared to 19% Ad35 seropositive among the Ad5 seropositive HIV-infected subjects. This difference does not reflect the lack of exposure to other adenovirus serotypes in the 70 HIV-infected subjects as the seroprevalence for other serotypes including Ad14, Ad28 and Ad41 in the Ad5 seropositive HIV-infected subjects is not significantly different from that in the HIV-uninfected Ad5 seropositives. It is quite possible that the infection rates were comparable but the HIV-infected cases did not generate a strong, long-lasting humoral response to a weakly immunogenic pathogen like Ad35. The data on magnitude of Ad5 titers and immune responses after vaccination and the correlations with subsequent HIV infection suggest that differences in immune function may predispose to infections seen in the Step study.

We also document differential effects of baseline Ad5 serostatus on CD4 and CD8 immune responses stimulated by the MRKAd5 HIV vaccine. The blunting effects of pre-existing Ad5 immunity were more apparent on the magnitude of CD8 responses than on CD4 responses. In the case of specific responses to Gag and Pol, Ad5 serostatus only affected CD8 responses to these two antigens. On the other hand, Ad5 serostatus reduced both CD4 and CD8 responses to Nef. Such differential effects on immune responses to different antigens were observed in DNA/rAd5 HIV vaccine trials: Ad5 serostatus had no effect on immune responses to HIV Env but affected anti-Gag immune responses [5]. Such blunting can affect vaccine immunogenicity in Ad5 seropositives and the boosting effect of vaccinations after the first in Ad5 seronegatives. Thus, a heterologous prime and boost regimen may further improve vaccine efficacy even in Ad5 seronegative subjects.

In summary, this study shows that the MRKAd5 HIV vaccine generated neutralizing antibodies to Ad5 hexon and penton capsid proteins more efficiently than to Ad5 fiber. Anti-vector immunity differed qualitatively in participants who acquired HIV infection compared to those who did not. This effect was independent of vaccination and may have arisen in part because of differences in the immune function of subjects enrolled from distinct geographic regions or HSV seropositivity or infection by other viruses. The increased HIV infection rates in Step were therefore confounded by these underlying increased risk factors and not clearly related to the vaccine. Pre-vaccination serostatus affected post-vaccination neutralizing antibody profiles and vaccine-induced immune responses to HIV antigens. These data also suggest that Nab to Ad5 fiber contributes to the reduction of vaccine potency. Thus, strategies to overcome pre-existing Nab must avoid the use of Ad5 fiber for vector construction.

## Materials and Methods

### Construction and Production of Adenovirus Vectors

The construction and propagation of the rAd5 and rAd35 vectors with wild-type capsid proteins and with chimeric fiber were previously described [21]–[23]. Total particle unit titer was determined by absorbance [24]. The transduction activity of these vectors was evaluated by titration on A549 cells to determine the sensitive range of MOI that can be used in neutralizing assays [5].

### Clinical Trials and Serum Samples

The Step trial is a Phase IIB, placebo-controlled trial with 3000 participants who received three intramuscular injections of either rAd5 HIV vaccine expressing HIV-1 gag/pol/nef or the vaccine diluent at day 0, week 4, and week 26 as previously described [1]. The trial was stopped due to lack of vaccine efficacy and futility. A trend towards increased HIV infection frequency was observed in vaccinated subpopulations, though it did not reach statistical significance.

To follow the development of anti-Ad5 neutralizing antibodies during vaccination, eighty-eight Step trial serum samples were received from the HVTN. These samples were from 11 randomly selected Ad5 seronegative and 11 randomly selected Ad5 seropositive participants collected on vaccination Day 0 and following each of the three MRKAd5 vector administrations (week 4, week 8, and week 30). The majority of the 22 participants were from North America (US or Canada, 68%) and the rest were from the Caribbean (Haiti or Puerto Rico, 18%) or South America (Peru, 14%).

To analyze the effect of pre-existing anti-Ad5 neutralizing antibodies on vaccine immunogenicity, human serum samples from 179 Step participants in the vaccine group were analyzed and immunogenicity data from these participants as measured by ICS assay at Week 30 were obtained from HVTN. Among the 179 participants, 51 were Ad5 seronegatives selected randomly. The rest were Ad5 seropositive participants at Day 0 and the titers of anti-Ad5 neutralizing antibodies in these participants were distributed evenly among low, mid and high ranges as previously determined by HVTN [2]. The majority of the participants were from North America (USA or Canada, 60%), the rest of them were from South America (Peru or Brazil, 22%), the Caribbean (Haiti or Dominican Republic, 17%) or Australia (1%).

71 Step trial serum samples collected on vaccination Day 0 from Ad5 seropositive participants who acquired HIV post-vaccination were also analyzed to reveal particular features of their anti-Ad5 neutralizing antibody profiles. These were all the HIV positives in the Step trial at the time of analysis. 64 of the participants were male with 30 circumcised, 32 uncircumcised, 2 unknown. The majority of them were from North America (USA or Canada, 59%), the rest were from South America (Peru or Brazil, 36%) or the Caribbean (Haiti or Dominican Republic, 5%).

### Neutralization of Adenovectors with Human Sera

The method for analyzing the neutralization of adenovectors by human sera was developed based on procedures published previously [5]. The sera were inactivated by heating at 56°C for 60 min and the inactivated sera were diluted with 10% FBS containing RPMI medium and mixed with the indicated rAd vector encoding luciferase for 30 min at room temperature for neutralization. To prepare A549 cell suspensions for transduction, cells grown in a 75 cm^2^ flask were harvested by treatment with 4 mM EDTA and suspended in 10% FBS containing RPMI medium. The neutralized virus was used to transfect A549 cells at 100 PU/cell, and luciferase expression was analyzed using the luciferase assay kit (Promega, Inc.) at 24 h post-transduction. The serum at the dilution of 1∶18 that reduced viral transduction activity by more than 90% was defined as seropositive [1]. The neutralization titer for such seropositive sera was also determined and defined as the maximum dilution that can reduce the viral transduction by 90% [25] as calculated using GraphPad Prism 5 software. A typical neutralization graph is shown in Figure S2.

### Analysis of T-cell Responses Post-vaccination

At 30 weeks after the first vaccination (four weeks post-third rAd vaccination), the T-cell responses to individual HIV-1 vaccine-matched peptide pools were analyzed according to previously published methods by an intracellular cytokine staining (ICS) assay [2]. The percentages of CD4+ and CD8+ T cells producing cytokines were reported with background correction. ICS data for some of the participants were not available at the time of analysis and the numbers of available data were noted in each figure and table.

### Statistical Analysis

The Nab titer and the magnitude of CD4+ or CD8+ T-cell immune responses were compared between seropositive and seronegative subjects using two-sided Wilcoxon Rank-Sum tests. These non-parametric tests were chosen due to the presence of large influential points, and the moderate sample sizes.

## Supporting Information

Figure S1 **The titers of Nab to Ad5, Ad5 F35, Ad35, and Ad35 F5 were determined in HIV-infected participants in the placebo (n = 26) and vaccine group (n = 44).** Geometric mean and 95% CI are shown in the background of Nab titers in each individual.
(PDF)

Figure S2 **Neutralizing activity of a representative human serum (participant ID 13958) against rAd is shown.** This serum contained high titers of neutralizing antibodies to Ad5, Ad5 F35 and Ad35 F5, with minimal Ad35 neutralizing antibodies.
(PDF)
