# Correction: Decreased Pre-existing Ad5 Capsid and Ad35 Neutralizing Antibodies Increase HIV-1 Infection Risk in the Step Trial Independent of Vaccination

**DOI:** 10.1371/annotation/805c32e9-70cf-4937-af15-e26de6a11e99

**Published:** 2012-05-31

**Authors:** Cheng Cheng, LingShu Wang, Jason G. D. Gall, Martha Nason, Richard M. Schwartz, M. Juliana McElrath, Steven C. DeRosa, John Hural, Lawrence Corey, Susan P. Buchbinder, Gary J. Nabel

The second author's name was spelled incorrectly. The correct name is: Lingshu Wang.

