# Supplementary figures and images for: Decreased Pre-existing Ad5 Capsid and Ad35 Neutralizing Antibodies Increase HIV-1 Infection Risk in the Step Trial Independent of Vaccination
Source: PLoS One. 2012 Apr 4;7(4):e33969. doi: 10.1371/journal.pone.0033969 (PMC3319553; doi:10.1371/journal.pone.0033969)

Figure S1

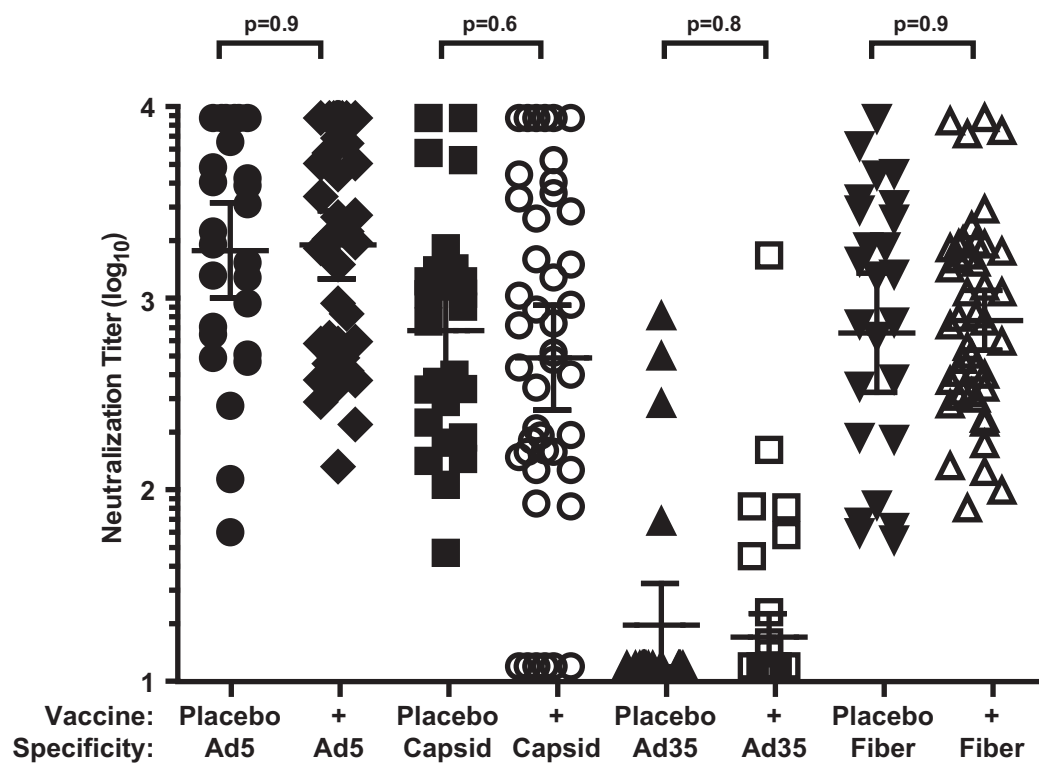

Supplement: Figure S1 — The titers of Nab to Ad5, Ad5 F35, Ad35, and Ad35 F5 were determined in HIV-infected participants in the placebo (n = 26) and vaccine group (n = 44). Geometric mean and 95% CI are shown in the background of Nab titers in each individual. (PDF) [file pone.0033969.s001.pdf]

Figure S2

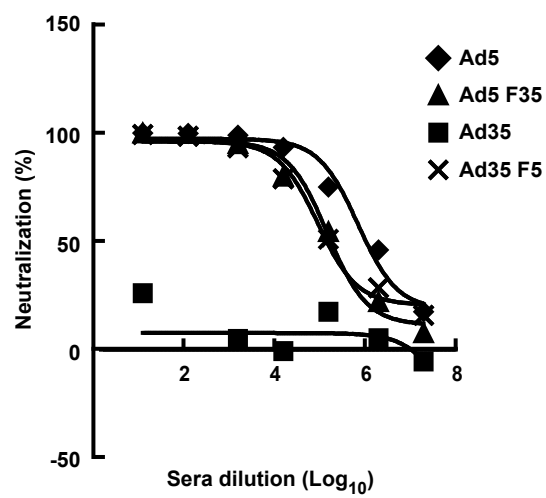

Supplement: Figure S2 — Neutralizing activity of a representative human serum (participant ID 13958) against rAd is shown. This serum contained high titers of neutralizing antibodies to Ad5, Ad5 F35 and Ad35 F5, with minimal Ad35 neutralizing antibodies. (PDF) [file pone.0033969.s002.pdf]
